# Required Levels of Catalysis for Emergence of Autocatalytic Sets in Models of Chemical Reaction Systems

**DOI:** 10.3390/ijms12053085

**Published:** 2011-05-12

**Authors:** Wim Hordijk, Stuart A. Kauffman, Mike Steel

**Affiliations:** 1 Department of Ecology and Evolution, University of Lausanne, 1015 Lausanne, Switzerland; 2 Tampere University of Technology, Finland; E-Mail: stukauffman@gmail.com; 3 University of Vermont, 85 South Prospect Street Burlington, VT 05405, USA; 4 Santa Fe Institute, 1399 Hyde Park Road Santa Fe, NM 87501, USA; 5 Department of Mathematics and Statistics, University of Canterbury, Private Bag 4800, Christchurch, New Zealand; E-Mail: mike.steel@canterbury.ac.nz

**Keywords:** autocatalytic sets, chemical reaction networks, template-based catalysis, origin of life

## Abstract

The formation of a self-sustaining autocatalytic chemical network is a necessary but not sufficient condition for the origin of life. The question of whether such a network could form “by chance” within a sufficiently complex suite of molecules and reactions is one that we have investigated for a simple chemical reaction model based on polymer ligation and cleavage. In this paper, we extend this work in several further directions. In particular, we investigate in more detail the levels of catalysis required for a self-sustaining autocatalytic network to form. We study the size of chemical networks within which we might expect to find such an autocatalytic subset, and we extend the theoretical and computational analyses to models in which catalysis requires template matching.

## 1. Introduction

In previous work we introduced and investigated a mathematical model of catalytic reaction systems and autocatalytic sets [1,2]. It was shown, both theoretically and computationally, that a linear growth rate in the level of catalysis (with increasing length *n* of the largest molecules in the system) is sufficient for autocatalytic sets to arise spontaneously [2,3] in a well-known binary polymer model of catalytic reaction systems [4,5].

In this paper we take a closer and more detailed look at our model and its results. First, we introduce a small modification to our mathematical definition of autocatalytic sets and the corresponding algorithm for finding them in general catalytic reaction systems (Section 3). This modification makes both the definition and the algorithm slightly simpler, and includes some specific (although probably rare) cases of autocatalytic sets which were previously left out. However, we show (formally, and in simulations) that this modified algorithm does not invalidate any previous results or conclusions.

Second, we show that there is a discrepancy between the theoretical and simulation results (Section 4). Both results show that a linear growth rate in level of catalysis is sufficient for the emergence of autocatalytic sets. However, there is a difference in the parameter values of these linear relations. Here, we recalculate and compare the required levels of catalysis in more detail and under different scenarios.

Third, we show how our model and algorithm can be used to answer other interesting questions relating to the emergence of autocatalytic sets (Section 5). In particular: What is the minimum required size of the molecule set for autocatalytic sets to emerge given a fixed (known) probability of a molecule catalyzing an arbitrary reaction?

Fourth, we show how more chemical realism can be included in our model, for example by considering template-based catalysis (Section 6). Even though this makes the model harder to analyze, it still generates interesting and useful results.

The next section briefly reviews our previously introduced model and definitions. The four sections following it will present the model modifications, extensions, and additional results mentioned above. The final section summarizes the main conclusions and discusses future directions. Mathematical proofs are provided in an Appendix.

Our study fits within a large and growing body of work that aims to formally model how self-sustaining biochemical systems necessary for life might have emerged. This is an area that has been investigated from many angles over the last three decades. Some approaches that are similar in scope but different in their specific details from the one we study here include models based on Petri-nets [6], algebraic approaches based on metabolic closure (such as Rosen’s “(M,R) systems”, [7–9]), computer simulations of autocatalytic networks involving artificial chemistry [10] or metabolic networks [11], differential equation modeling [12–15], and Erdös-Renyi style random graph theory [16]. The idea of autocatalytic sets as a precursor to life has certainly not been without criticism [17–19], but recent (exciting) experimental evidence shows that they are a real possibility [20–24]. Thus, we believe that our theoretical and computational studies and results are of direct relevance in the larger context of the origin of life [5,25,26].

## 2. RAF Sets

Autocatalytic sets may have played an important role in the origin of life [5,15,26,27], and are a necessary, although not sufficient, condition for life. Here, *autocatalytic sets* are defined more formally as *RAF sets* as follows (see [1,2] for the full mathematical definition and notations). Given a *catalytic reaction system* (CRS), *i.e.*, a network of molecule types and catalyzed chemical reactions, a (sub)set *ℛ* of such reactions (plus the molecules involved in the reactions in *ℛ*) is called:

1. *Reflexively autocatalytic* (RA) if every reaction in *R* is catalyzed by at least one molecule involved in any of the reactions in *ℛ*;
2. *F-generated* (F) if every reactant in *ℛ* can be constructed from a small “food set” *F* by successive applications of reactions from *ℛ*;
3. *Reflexively autocatalytic and F-generated* (RAF) if it is both RA and *F*.

**Figure 1.**
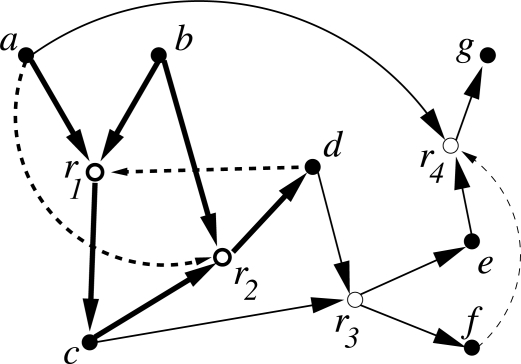
A simple example of a *catalytic reaction system* (CRS) with seven molecule types {*a, b, c, d, e, f, g*} (solid nodes) and four reactions {*r*_1_, *r*_2_*, r*_3_*, r*_4_} (open nodes). The food set is *F* = {*a, b*}. Solid arrows indicate reactants going into and products coming out of a reaction, dashed arrows indicate catalysis. The subset *ℛ* = {*r*_1_*, r*_2_} (shown with bold arrows) is RAF.

The food set *F* contains molecules that are assumed to be freely available in the environment. Thus, an RAF set formally captures the notion of “catalytic closure”, *i.e.*, a self-sustaining set supported by a steady supply of (simple) molecules from some food set. Figure 1 shows a simple example. In [2], we also introduced a polynomial-time algorithm to find RAF sets in general catalytic reaction systems.

Note (as already stated earlier [26]) that this notion of an autocatalytic *set* is somewhat different from the (chemical) term autocatalytic *reaction* in which a molecule directly catalyzes its own production. With an autocatalytic set we do *not* mean a set of autocatalytic reactions, but rather a set of molecules and reactions which is collectively autocatalytic in the sense that all molecules help in producing each other (through mutual catalysis, and supported by a food set). Because of this confusion in terminology, we prefer to use the term “RAF set”.

### 

#### A Model of Catalytic Reaction Systems

In [4,5], a random CRS model was introduced using binary polymers (bit strings) of length at most *n* as molecule types, ligation and cleavage reactions, and a probability *p* of any molecule catalyzing any reaction (*n* and *p* are parameters of the model). It was argued that in a “sufficiently complex” system (*i.e.*, large enough diversity of molecule types), autocatalytic sets would appear “spontaneously”. This was later criticized for requiring an exponential increase (with *n*) in the (average) number of reactions catalyzed by any one molecule [17], which is chemically unrealistic. However, in [2] it was shown computationally (by applying the RAF algorithm to instances of the random CRS model), and then confirmed theoretically in [3], that only a *linear* growth rate (with *n*) in the level of catalysis is sufficient for RAF sets to appear with high probability. Furthermore, with these results it is possible to quantify “sufficiently complex” in terms of the size of the molecule set (or maximum molecule length *n*) and the level of catalysis (average number of reactions catalyzed by any molecule). Here, we continue our investigations of this random CRS model, which we will refer to throughout as the *binary polymer model*.

## 3. Modification of the Original Definition and Algorithm

In the original (mathematical) definition of RAF sets [2], the “RA” part is stated in terms of the *support* supp(*ℛ*) of a set of reactions *ℛ*, which is defined as all the molecules (reactants and products) that are involved in at least one of the reactions in *ℛ*. For a set of reactions *ℛ* to be reflexively autocatalytic (RA), it is required that every reaction in *ℛ* is catalyzed by at least one molecule in supp(*ℛ*).

Similarly, the “F” part of the definition is stated in terms of the *closure* cl*_ℛ_*(*F*) of the food set *F* relative to the set of reactions *ℛ*, which is defined as *F* together with all molecules that can be constructed from *F* by repeated applications of reactions in *ℛ*. For a set of reactions *ℛ* to be *F*-generated (*F*), it is required that the reactants of every reaction in *ℛ* are in cl*_ℛ_*(*F*).

Now imagine a situation where all reactions in *ℛ* conform to the above two requirements, except for one reaction *r* ∈ *ℛ* which is catalyzed by a molecule *x* ∈ *F* which is not involved in any other way in any reaction in *ℛ*. So, *x* ∈ cl*_ℛ_*(*F*), but *x* ∉ supp(*ℛ*). In words, the molecule *x* is in the closure of the food set (by default, as it is part of the food set), but it is not in the support of the reaction set. So, according to the original definition, this set *ℛ* is not RAF (it does not conform to the RA part of the definition), even though logically one would consider this case to be a proper autocatalytic set.

To remedy this, and make sure these (probably rare but relevant) cases are also included, we propose a slight modification to our original definition of RAF sets as follows:

### 3.1. RAF Definition

Given a catalytic reaction system *𝒬* = (*X, ℛ, C*), with molecule set *X*, reaction set *ℛ*, catalyzation *C* (a set of molecule-reaction pairs indicating which molecules catalyze which reactions), a nonempty subset *ℛ′* of *ℛ* is said to be:

- *Reflexively autocatalytic* (RA) if for all reactions *r* ∈ *ℛ′* there exists a molecule *x* ∈ cl*_ℛ′_* (*F*) such that (*x, r*) ∈ *C*;
- *F-generated* if ρ(*ℛ′*) ⊆ cl*_ℛ′_* (*F*), where ρ(*ℛ′*) is the set of all reactants in *ℛ′;*
- *Reflexively autocatalytic and F -generated (RAF)* if *ℛ′* is both RA and *F*.

In other words, a set of reactions *ℛ′* is RAF if each reaction in *ℛ′* is catalyzed by at least one molecule that can be produced by the set itself (starting from the food molecules), and all reactants (of all reactions in *ℛ′*) can also be produced by the set itself. So, instead of using supp(*ℛ*) in the RA part of the definition and cl*_ℛ_*(*F*) in the F part, as in the original definition, the modified definition uses cl*_ℛ_*(*F*) in both the RA and the F part, thus simplifying it slightly. Note that an RAF in the original setting of [2] is still an RAF under this new definition by virtue of the following result, a proof of which is provided in the Appendix.

**Lemma 3.1** *Any set of reactions that forms an RAF under the earlier definition of [2] is an RAF in the modified definition above.*

This may seem like a minor point, but it could be an important one. Consider, for example, the (reverse) citric acid cycle, which has been argued to have (possibly) been a major step in the origin of life by synthesizing the basic building blocks of organic molecules [28,29]. Even though at present the catalysts that drive the reactions in this cycle are enzymes (proteins), at the early stages these reactions could very well have been catalyzed by much simpler, naturally occurring elements, or ones that are easily synthesized (by basic chemistry) from freely available inorganic molecules [30]. Thus, these original catalysts can be considered food molecules, but they are not involved (as reactants or products) in any of the reactions in the (reverse) citric acid cycle (*i.e.*, they are not in the support). As a consequence, this important metabolic network of reactions would not be classified as an RAF with our original definition, but it is indeed a true RAF according to our modified definition.

Lemma 3.1 has several other desirable properties. First, it ensures that the recent result that Rosen’s (M,R) systems can be viewed and studied within the RAF framework [9], still holds. Second, it tells us that if a system has no RAF under the new definition, then it clearly has none under the original one, nor, indeed, under more embellished definitions of an RAF that impose further conditions so as to avoid “trivialities”. One such condition (from [1]) would be to require that not all reactions in an RAF are catalyzed by molecules in *F* (or in some larger subset *S* of molecules that can be generated from *F* by catalysis from *F* and resulting molecules). However, we can accommodate this additional condition within the new RAF framework as follows. The algorithm we will describes below constructs a unique maximal RAF (under the new definition), and from this one can easily check whether an RAF exists that satisfies the additional non-triviality condition (simply check whether this maximal RAF contains a reaction that does not have all its catalysts in *F*, or the larger set *S*). Third, spontaneous reactions (those that can proceed without any catalyst) can be allowed (and form part of an RAF) if we formally extend *F* by an extra “token” element with directed (catalysis) arrows from that token to all such spontaneous reactions. And there are various other extensions, restrictions, or variations that can be imposed on our more general framework.

Of course there are many more properties relevant to the origin of self-sustaining biochemistry beyond such ad-hoc conditions to exclude trivialities—for example, the dynamics of the reactions (the quantity of reagents and products (stoichiometry) along with thermodynamic considerations) and the effect of inhibition and degrading side reactions [18,19]. However, our view here is that RAF sets should be regarded very much as minimal *necessary* conditions for such systems, rather than in any sense *sufficient*. Identifying RAF sets, and establishing conditions for their existence is thus a natural question on the road to finding viable candidates for the origin of early biochemistry.

An additional benefit of our modified RAF definition is that it significantly simplifies the corresponding algorithm for finding RAF sets in general catalytic reaction systems, and its correctness proof. The original algorithm is based on repeatedly (and alternately) applying two reduction steps (starting from the full reaction set) [2]:

1. Remove all reactions that do not conform to the RA requirement;
2. Remove all reactions that do not conform to the *F* requirement.

However, since in our modified definition both the RA part and the *F* part are stated in terms of the closure, these two reduction steps can now be merged into one. As a consequence, the algorithm can be simplified to the following:

### 3.2. RAF Algorithm

1. Start with the complete set of reactions *ℛ* and the food set *F*;
2. Compute the closure of the food set cl*_ℛ_*(*F*) relative to the current set of reactions *ℛ*;
3. For each reaction *r* ∈ *ℛ* for which (1) all catalysts, or (2) one or more reactants, are not in cl*_ℛ_*(*F*), remove *r* from *ℛ*;
4. Repeat steps 2 and 3 until no more reactions can be removed.

The resulting (reduced) reaction set *ℛ* is either the (maximal) RAF set contained in the given catalytic reaction system, or it is empty, in which case there is no RAF. This was already proved for the original algorithm in [2], and a (simpler) proof for the modified version is provided in the appendix. Note, however, that the overall running time of this modified algorithm is still the same as in the original case (*𝒪*(|*ℛ*|^2^ log |*ℛ*|) worst-case, but shown to be sub-quadratic on average in practice [2]), as it is dominated by step 2 (computing the closure of the food set). All results presented in this paper are generated with this new version of the RAF algorithm.

## 4. Required Levels of Catalysis

In [2], we showed through computer simulations that a linear growth rate in the level of catalysis (with *n*, the length of the largest molecules in the system) appears to be sufficient for RAF sets to occur with high probability in the binary polymer model with ligation and cleavage reactions and random catalysis. This was subsequently confirmed theoretically in [3]. However, even though both the computational and theoretical results give a linear relation for the required level of catalysis, there appears to be a significant difference in the values of the parameters of these linear functions.

This discrepancy can partly be explained by the fact that the theoretical analysis in [3] actually assumes RAF sets that involve *all* molecule types in the system, *i.e.*, an RAF that contains the entire molecule set *X* (but not necessarily all reactions). This, of course, is a much stronger assumption than used in the simulation studies in [2], where the RAF algorithm was used to find *any* RAF set, regardless of how many molecules or reactions it contains. But the question remains whether the discrepancy can be explained entirely by this difference.

To answer this, we repeated the original simulations, using the RAF algorithm to find *any* RAF set, but this time with the modified RAF algorithm. Then we also applied the RAF algorithm to look for RAF sets that involve *all* molecule types in *X*. In both cases, we collected statistics for the average number *f* (*n*) of reactions catalyzed by any molecule (*i.e.*, the level of catalysis) for which there is a probability *P_n_* = 0.50 (or close to 0.50) of finding an RAF set in a number of instances of the random catalytic reaction model [31]. From these statistics, we then estimated a linear function *f* (*n*) = *a* + *bn* using an ordinary least squares regression. We compare these results with the theoretical linear relation which can be calculated from Theorem 4.1 (ii) in [3] (using *P_n_* = 0.50 and *k* = *t* = 2).

So, in short, we compare the required levels of catalysis for RAF sets to occur with high probability for three cases:

- Computational case for *any* RAF;
- Computational case for *all-molecule* RAFs;
- Theoretical case for *all-molecule* RAFs.

The computational values were calculated over 100 to 1000 (depending on the value of *n*) instances of the random catalytic reaction model, for *n* = 7, . . ., 20 (because of an exponential increase in the number of molecules |*X*| and reactions |*ℛ*| with *n*, we are computationally limited to about *n* = 20 in these simulations).

Table 1 presents the linear relations estimated from the simulation data or calculated from the theoretical analysis. There is a difference of almost 2 orders of magnitude between the observed (simulation) slope of the required growth rate (case **A**) and the theoretical one (case **C**). However, as case **B** shows, this difference cannot be fully explained by the fact that the theoretical analysis assumes RAFs involving *all* molecules. Even with this stronger assumption, the theoretical slope (case **C**) is still more than twice as large as the one from the simulations (case **B**).

**Figure 2.**
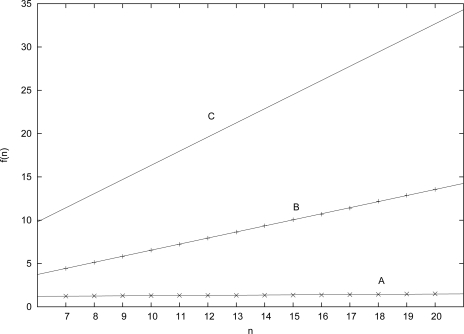
The linear relations for the three cases.

Figure 2 shows the complete data (dots for simulation values, lines for estimated or calculated linear relations). Clearly, even though the theoretically predicted value for *f* (*n*) grows quite fast with *n* (slope = 1.6339), the actual value grows only very slowly (slope = 0.0189). So, for example, for *n* = 20 the theoretically expected value for the average number of reactions that need to be catalyzed by any molecule to have RAF sets occurring with probability at least *P_n_* = 0.50 is *f_C_* (20) = 32.678, which seems unrealistically high. However, the actual level of catalysis required is only *f_A_*(20) = 1.475, which is chemically much more plausible.

## 5. Required Size of the Molecule Set

In addition to the level of catalysis required for RAF sets to emerge, we can use the RAF algorithm to answer other interesting, and related, questions. For example, one could ask what the minimum required size of the molecule set is (or, in the binary polymer model, the minimum size *n* of the largest molecules in the system) to get RAF sets with high probability, given a fixed (known) probability *p* of a molecule catalyzing an (arbitrary) reaction.

Suppose we fix the probability of catalysis at *p* = 0.00001, or perhaps even *p* = 0.000001 (one in a million), which is, for example, roughly the probability in phage display of a random peptide binding an arbitrary ligand [5,32], and “the ease of polypeptide evolution with a small number of arbitrary sequences indicates that a significant fraction of all possible sequences may have functions, at least binding activity in correlation with catalytic activity” [33]. In the binary polymer model, with these probabilities of catalysis, what value of *n* is required to get a probability of, say, *P_n_* ≥ 0.50 of RAF sets occurring?

**Figure 3.**
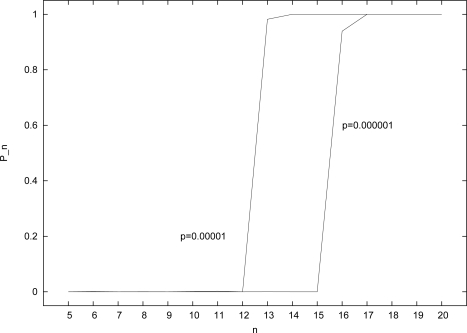
The probability *P_n_* of finding RAF sets for different (fixed) catalysis probabilities *p* and values of *n*.

Figure 3 shows the results of applying the (modified) RAF algorithm to instances of the random binary polymer model (Section 2) for the two given values of *p* and for *n* = 5, . . ., 20. With *p* = 0.00001 (left curve in Figure 3), *P_n_* = 0 for *n ≤* 12, but *P*_13_ = 0.982, and *P_n_* = 1 for *n* ≥ 14. So, in this case a value of at least *n* = 13 is required to get RAF sets with high probability. For only a slightly higher probability of catalysis (*p* = 0.00002), a value of *n* = 12 would be sufficient (results not shown).

Similarly, with *p* = 0.000001 (right curve in Figure 3), *P_n_* = 0 for *n ≤* 15, *P*_16_ = 0.939, and *P_n_* = 1 for *n ≥* 17. So, in this case a value of at least *n* = 16 is required to get RAF sets with high probability. Again, for only a slightly higher probability of catalysis (*p* = 0.000002), a value of *n* = 15 would be sufficient (results not shown). The size of the molecule set in this case would be |*X*| = 65534.

## 6. Template-Based Catalysis

One could argue that the binary polymer model used in our studies so far is perhaps somewhat oversimplified to be biologically or chemically realistic. However, the model serves as a useful starting point with which precise mathematical statements can be formulated and proved, or at least tested computationally. Furthermore, our RAF definition and algorithm are independent of the particular model that is used, and can in principle also be applied to real catalytic reaction systems (for example metabolic networks, of which the already mentioned citric acid cycle is a core element). And, equally importantly, it is actually not very difficult to add more chemical realism into our mathematical models.

As one particular example, we have considered *template-based catalysis* [5]. The idea here is that, in order to act as a catalyst, a molecule must match at least a certain area around the reaction site according to some template-based matching rule. Similar to, for example, base-pair complementarity in RNA and DNA, we could require a catalyst to match the complement of at least four positions around the reaction site of a ligation or cleavage (two on either side). Consider the ligation reaction

00101 + 0011 −> 001010011

The reaction site template in this case is 0100 (the last two bits of the first binary polymer, plus the first two bits of the second polymer). A given molecule can only act as a catalyst for this reaction if, somewhere along its binary string representation, it contains the complement of this template, *i.e.*, 1011.

In line with the original random CRS model, and some initial simulations with such template-based catalysis [4,5], we have included this idea as follows. For each combination of a molecule *x* ∈ *X* and a reaction *r* ∈ *ℛ*, if *x* matches (anywhere along its length) the complement of the reaction site template of length four of *r*, then with probability *p* the pair (*x, r*) is included in the set of catalyzation *C*. We present analytical results for this model in Section 6.1 below. However, in our simulations (Section 6.2 below), we used a slightly less constrained version as follows: If a molecule happens to be shorter than length four, then it only has to match part of the complement of the reaction site template, but we require catalysts to be of at least length two. So, the molecule 01 could also catalyze the reaction in the above example. This is mainly done to give some of the food molecules (which are all bit strings up to length two in our simulations) also a chance to act as catalysts.

Note that this template matching requirement is almost the same as in the original simulations [5,34–36], except that we do not allow partial matches here. So, we are considering a slightly “stronger”, or more constrained case. Some initial results on similar simulations were reported in [37].

### 6.1. Theoretical Results

Theorem 4.1 (ii) of Mossel and Steel (2005) shows that for polymers of length up to *n* over an alphabet of size *κ* ≥ 2, and with a food set of polymers of length up to *t*, the probability *P* (*n*) that there exists an all-molecule RAF is at least:

$$P\left(n\right)\;\geq \;1\;-\;\frac{\kappa {\left(\kappa e^{-\lambda }\right)}^{t}}{1\;-\;\kappa e^{-\lambda }}$$

when each molecule catalyzes on average (at least) *λn* reactions (provided also that *λ* > log*_e_*(*κ*)). Notice that *P* (*n*) can be chosen as close to 1 as we wish by selecting *λ* large enough (and independently of *n*!). Thus, this result justifies the statement that the average number of reactions each molecule catalyzes needs to grow only linearly with *n* in order for there to be a given (high) probability of generating an RAF.

We now describe how this result modifies if catalysis is required to be template-based, as described above. Suppose that a polymer *x* can catalyze a given reaction only if *x* contains a substring of length *s* = *s*_1_ + *s*_2_ that is complementary to the end-segment (of length *s*_1_) and the initial segment (of length *s*_2_) of the two molecules involved in the cleavage or ligation. Thus, for the above set-up we have: *s*_1_ = *s*_2_ = 2 and so *s* = 4. We also assume that the probability that a molecule *x* catalyzes a reaction *r* with a complementary template just depends on *x* and not on *r* (this is the analogue of the template-free model assumption (*R*_2_) in [3]).

The following result shows that RAFs will still arise with high probability under linear growth in the average number of reactions each molecule catalyzes, provided the constant involved is increased by a factor of *κ^s^* (this factor would be 16 for the binary model with template size four). More precisely we have the following result, whose proof is provided in the Appendix.

**Theorem 6.1** *Let P_s_(n) be the probability that there exists an all-molecule RAF under this template matching model. Suppose that each molecule catalyzes (on average) at least λ_s_n reactions, where λ_s_* *= κ^s^λ and λ >* log*_e_(κ). Then P_s_(n) satisfies the same inequality as P (n), namely*

$$P_{s}\left(n\right)\;\geq \;1\;-\;\frac{\kappa {\left(\kappa e^{-\lambda }\right)}^{t}}{1\;-\;\kappa e^{-\lambda }}$$

### 6.2. Computational Results

As with the original (random) model (see Section 4), we expect there to be a difference between the theoretically predicted required level of catalysis and the observed level (from simulations) in case of template-based catalysis. Unfortunately, we are computationally even more restricted with this template-based catalysis case as with the original model. The running time of our RAF algorithm is polynomial in the size of the reaction set |*ℛ*|, as was shown in [2]. However, since |*ℛ*| is *exponential* in the size of the largest molecules *n*, *i.e.*, |*ℛ*| ∝ 2*^n^*, we can only go up to about *n* = 20 to get computational results in a reasonable amount of time (even on a parallel cluster) for the original model (Section 4). But for the template-based catalysis as described above, it is even worse. We now have to check for each pair of molecule *x* ∈ *X* and reaction *r* ∈ *ℛ* whether there is a template match between any part of the molecule and the complement of the (4-site) reaction template. Since both *|X|* and *|ℛ|* are ∝ 2*^n^*, this means that |*X* × *ℛ*| ∝ 2^2^*^n^*, and in practice this means that we can only go up to about *n* = 16 with our template-based catalysis simulations.

**Figure 4.**
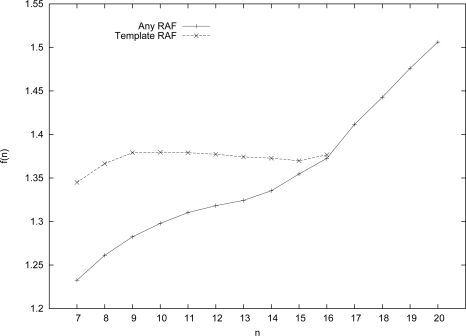
The level of catalysis *f* (*n*) required for the template-based catalysis case compared to the original (purely random) case, for different values of *n*.

Figure 4 shows the results of these simulations, using our modified algorithm to find RAF sets (of any size) in the template-based catalysis case, compared to the original model (case **A** in Section 4). As can be expected, for smaller values of *n*, a higher level of catalysis is needed to find RAF sets with high probability (again, *P_n_* = 0.50 is taken as the transition point) in the case of template-based catalysis compared to the purely random model. Since each molecule type *x* ∈ *X* is now restricted, to some extent, in terms of which reactions it can catalyze, the system as a whole is more constrained, and it will be harder to get RAF sets.

However, for longer and longer molecule types, this restriction becomes less of a problem, as a longer molecule has an increasingly higher probability of matching a given 4-site template somewhere along its length. Indeed, the required level of catalysis for the template-based case tapers off as *n* increases, and converges to that of the original (purely random) model, reaching the same level at *n* = 16. Given that the purely random model is the “limiting” case for the template-based model, we expect that the level of catalysis for this template-based model will follow the same pattern as the base model for *n >* 16, and follow a linear relation.

## 7. Conclusions

Building on our previous work, we have investigated in more detail the levels of catalysis required for the emergence of autocatalytic sets in models of chemical reaction systems. First, we have shown that there is a discrepancy between the theoretically predicted levels and the computationally observed ones. Although both results yield a linear relation between the required level of catalysis and the size *n* of the largest molecules in the system, in practice this required level is almost two orders of magnitude smaller than the predicted one. However, this discrepancy cannot be fully explained by the fact that the theoretical result is based on a much stronger assumption (requiring the RAF sets to contain *all* molecules in the system). Even for large systems (*n* = 20, containing several millions of molecule types), each molecule only needs to catalyze (on average) between one and two reactions to have RAF sets appear with high probability, which is chemically highly plausible.

Next, we looked at the minimum size of the molecule set (*i.e.*, number of different types of molecules) necessary to get RAF sets given a fixed probability of catalysis. With a (realistic) probability on the order of one in a million of any molecule catalyzing any given reaction, we only need molecules up to length *n* = 15 or *n* = 16, or about 65,000 different molecule types. Again, this number is well within a plausible (experimental) range.

Finally, we studied an extension of the original model, including template-based catalysis. We established formally that in this case a linear growth rate in the level of catalysis also suffices for RAF sets to appear with arbitrary high probability. However, the simulations show that for smaller values of *n* (the length of the largest molecules in the system), this linear relation is not exact, and that a higher level of catalysis is necessary compared to the original (purely random) model to get RAF sets with a similar probability. But, as *n* increases, the template matching constraint becomes less of an issue, and the required levels of catalysis converge to those of the original model. This example shows how more chemical realism can be included in our model.

We intend to continue studying the emergence of autocatalytic sets in chemical reaction systems under various scenarios, models, and extensions. However, so far we have mainly studied the (static) underlying graph structures of such systems. One particularly important issue we hope to address in the future is the actual molecular dynamics in a given (catalytic) reaction system. Also, next to studying models of chemical reaction systems, we would like to apply our RAF framework to real (bio)chemical systems such as metabolic networks, or the collection of all known (organic) substrates and reactions. It is our hope that this line of work will help provide more insight into the (possible) origin of life in general.

## Figures and Tables

**Table 1.** The empirical (cases **A** and **B**) and theoretical (case **C**) linear relations.

**A**	*f_A_*(*n*) =	1.0970 + 0.0189*n*
**B**	*f_B_* (*n*) =	–0.4736 + 0.7012*n*
**C**	*f_C_* (*n*) =	1.6339*n*

## Appendix

### A1. Proof of Lemma 3.1

Let RA denote the original definition of “Reflexively Autocatalytic” from [2], and [RA_1_] the new one as given here in Section 3. We need to show that [RA] + [F-gen] implies [RA_1_], so suppose that *r* ∈ *R′*. By [RA] there exists *x* ∈ *supp*(*ℛ′*) : (*x, r*) ∈ *C.* Either *x* ∈ *ρ*(*R′*) or *x* ∈ *π*(*R′*) (the molecules that are products of at least one reaction in *ℛ′*). In the first case, by [F-gen], we have *x* ⋲ cl*_ℛ′_* (*F*) so [RA_1_] holds. In the second case, where *x* ∈ *π*(*R′*) there exists (*A, B*) ∈ *R′* for which *x* ∈ *B*. By [F-gen], *A* ⊆ cl*_ℛ′_* (*F*) and so *B* ⊆ cl*_ℛ′_* (*F*) and thus *x* ∈ cl*_ℛ′_* (*F*). So once again [RA_1_] holds.

### A2. Proof of RAF Algorithm Correctness

Let *ℛ′* be the output of the RAF algorithm. We need to establish two claims, namely that (i) if *ℛ′* is empty then *ℛ* contains no RAF, and (ii) if *ℛ′* is nonempty then *ℛ′* is an RAF, and indeed the unique maximal RAF present in *ℛ*.

To establish claim (i) we first justify a third claim (iii): If a reaction *r* lies in any RAF (say *ℛ*_1_) that is contained within an arbitrary subset (say *ℛ*_2_) of *ℛ*, then *r* is not eliminated if step 3 of the RAF algorithm is applied with *ℛ*_2_ being taken as the “current set of reactions”. To see this, the fact that *ℛ*_1_ is an RAF implies that there exists a molecule *x* ∈ cl_*ℛ*_1__ (*F*) that catalyzes *r*, and that the reactants of *r* are contained within cl_*ℛ*_1__ (*F*). Now, *ℛ*_1_ ⊆ *R*_2_ and so cl_*ℛ*_1__ (*F*) ⊆ cl_*ℛ*_2__ (*F*); thus step 3 will not eliminate *r* from *ℛ*_2_. This establishes claim (iii) which implies, by induction, the further claim (iv): If a reaction *r* lies within any RAF that is contained within *ℛ*, then *r* is never eliminated at step 3 of the RAF algorithm at any stage starting with *ℛ*. Claim (i) now follows immediately.

To establish claim (ii) we first note that, since the algorithm has terminated at some non-empty set of reactions *ℛ′*, no reaction in *ℛ′* can be further eliminated by step 3. By definition, this means that each reaction *r* in *ℛ′* has the properties that (1) at least one catalyst of *r* is in cl*_ℛ′_* (*F*) and (2) all reactants of *r* are in cl*_ℛ′_* (*F*). Condition (1) implies that *ℛ′* satisfies the (RA) condition, and property (2) shows that *ℛ′* satisfies the F-generated condition. Thus *ℛ′* is an RAF; moreover it is the unique maximal RAF by virtue of claim (iv) established in the previous paragraph.

### A3. Proof of Theorem 6.1

We first recall two quantities (*x_m_, r_m_*) from [3]. Let

(1) $$x_{m}\;=\;\frac{\kappa^{m+1}\;-\;\kappa }{\kappa \;-\;1}$$

denote the number of polymers of length at most *m* over an alphabet of size *κ* and let *r_m_* denote the number of forward (ligation) reactions that produce polymers of length at most *m*. We have:

(2) $$r_{m}\;\leq \;mx_{m}$$

For any polymer *x* of length at most *n* let *T_s_*(*x*) denote the set of polymers of length *s* that occur within *x*, and for any polymer *t* of length *s* let *X_n_*(*t*) denote the set of polymers of length at most *n* that contain at least one copy of *t*. Thus,

$$t\;\in \;T_{s}\left(x\right)\;\Leftrightarrow \;x\;\in \;T_{n}\left(t\right)$$

Clearly,

(3) $$1\;\leq \;\left|T_{s}\left(x\right)\right|\;\leq \;\kappa^{s}$$

Also, for *n ≥ s*, and *t* any polymer of length *s* we have:

4 $$\left|X_{n}\left(t\right)\right|\;\geq \;1\;+\;\kappa \;+\;\kappa^{2}\;+\;\cdots \;+\;\kappa^{n-s}\;=\;\frac{\kappa^{n-s+1}\;-\;1}{\kappa \;-\;1}$$

Given a (forward) reaction *r*, let *t_r_* denote the complement of the template polymer of length *s* = *s*_1_ + *s*_2_ (which is also polymer of length *s*). If we let *R_n_*(*t*) denote the set of forward reactions *r* with *t_r_* = *t*, then:

(5) $$\left|R_{n}\left(t\right)\right|\;=\;r_{n-s}$$

In the template polymerization model, *x* can catalyze *r* only if *x* ∈ *X*(*t_r_*) (or, equivalently, if *r* ∈ *R_n_*(*t*) for some *t* ∈ *T_s_*(*x*)). Let *p*(*x, r*) be the probability that molecule *x* catalyzes reaction *r*. We have assumed that each molecule that could catalyze reaction *r* (by virtue of template matching) has the same probability of doing so, that is:

$$p\left(x,\;r\right)\;=\;\{\begin{array}{c} p_{x},\;\text{if}\;t_{r}\;\in \;T_{n}\left(x\right)\\ 0,\;\text{otherwise} \end{array}$$

where *p_x_* just depends on *x* and not *r* (the analogue of requirement (R2) in [3]).

Thus, if we let *μ_x_* be the expected number of (forward) reactions (*r* ∈ *R*_+_(*n*)) that a molecule *x* catalyzes, then:

(6) $$\mu_{x}\;=\;\sum_{r\in R_{+}\left(n\right)}p\left(x,\;r\right)\;=\;p_{x}\;\cdot \;\left|S\left(x\right)\right|$$

where *S*(*x*) = {*r* ∈ *R*_+_(*n*) : *x* ∈ *X* (*t_r_*)}. Note that *|S*(*x*)*|* is bounded above by the number of pairs (*t, r*) where *t* ∈ *T_s_*(*x*) and *r* ∈ *R*_+_(*n*) : *r_t_* = *t*. By virtue of Inequality (3) and Equation (5) this set of pairs has size at most *κ^s^r_n–s_* and so

(7) $$\left|S\left(x\right)\right|\;\leq \;\kappa^{s}\;r_{n-s}$$

Now, Inequality (2) applied to *m* = *n – s*, and (7) gives:

$$\left|S\left(x\right)\right|\;\leq \;\kappa^{s}\;r_{\left(n-s\right)}\;\leq \;\kappa^{s}\left(n\;-\;s\right)x_{n-s}$$

Thus, by the assumption that *μ_x_* ≥ *λ_s_n* and applying (6), we obtain:

$$\lambda_{s}\;\cdot \;n\;\leq \;\mu_{x}\;\leq \;p_{x}\kappa^{s}\;\left(1\;-\;s/n\right)x_{n-s}\;\cdot \;n$$

Since *λ_s_* = *κ^s^λ* this last inequality can be written as:

(8) $$p_{x}\;\geq \;\frac{\lambda }{\left(1\;-\;s/n\right)x_{n-s}}$$

Now, following [3], for a forward reaction *r* ∈ *R*_+_(*n*) let $q_{r}^{*}$ be the probability that *r* is not catalyzed by any molecule. Then

$$q_{r}^{*}\;=\;\prod_{x\in X\;\left(t_{r}\right)}\left(1\;-\;p\left(x,\;r\right)\right)\;=\;\prod_{x\in X\;\left(t_{r}\right)}\left(1\;-\;p_{x}\right)$$

By Inequalities (4) and (8) and the inequality (1 – *x*)*^y^* ≤ exp(–*xy*) for *x, y >* 0 we obtain:

$$q_{r}^{*}\;\leq \;\text{exp}\;\left(-\lambda \;\cdot \;\frac{\kappa^{n-s+1}\;-\;1}{\left(1\;-\;s/n\right)x_{n-s}\;\left(\kappa \;-\;1\right)}\right)\;<\;\text{exp}\left(-\lambda \right)$$

where the second inequality follows by replacing *x_n–s_* by the expression given by Equation (1) for *m* = *n* – *s* and checking that the ratio that post-multiplies *λ* is greater than 1. The remainder of the proof now follows the argument in [3] for the proof of Theorem 4.1 (ii).

## References

[1] Steel M (2000). The emergence of a self-catalysing structure in abstract origin-of-life models. Appl. Math. Lett.

[2] Hordijk W, Steel M (2004). Detecting autocatalytic, self-sustaining sets in chemical reaction systems. J. Theor. Biol.

[3] Mossel E, Steel M (2005). Random biochemical networks: The probability of self-sustaining autocatalysis. J. Theor. Biol.

[4] Kauffman SA (1986). Autocatalytic sets of proteins. J. Theor. Biol.

[5] Kauffman SA (1993). The Origins of Order.

[6] Sharov A (1991). Self-reproducing systems: Structure, niche relations and evolution. BioSystems.

[7] Letelier JC, Soto-Andrade J, Abarzúa FG, Cornish-Bowden A, Cárdenas ML (2006). Organizational invariance and metabolic closure: Analysis in terms of (M,R) systems. J. Theor. Biol.

[8] Cornish-Bowden A, Cárdenas ML, Letelier JC, Soto-Andrade J (2007). Beyond reductionism: Metabolic circularity as a guiding vision for a real biology of systems. Proteomics.

[9] Jaramillo S, Honorato-Zimmer R, Pereira U, Contreras D, Reynaert B, Hernández V, Soto-Andrade J, Cárdenas M, Cornish-Bowden A, Letelier J (M,R) Systems and RAF Sets: Common Ideas, Tools and Projections.

[10] Flamm C, Ullrich A, Ekker H, Mann M, Hoegerl D, Rohrschneider M, Sauer S, Scheuermann G, Klemm K, Hofacker IL (2010). Evolution of metabolic networks: A computational framework. J Syst Chem.

[11] Kun A, Papp B, Szathmáry E (2008). Computational identification of obligatorily autocatalytic replicators embedded in metabolic networks. Genome Biol.

[12] Awazu A, Kaneko K (2007). Discretness-induced transition in catalytic reaction networks. Phys Rev E.

[13] Brogioli D (2010). Marginally stable chemical systems as precursors of life. Phys Rev Lett.

[14] Bartsev SI, Mezhevikin VV (2008). On initial steps of chemical prebiotic evolution: Triggering autocatalytic reaction of oligomerization. Adv. Space Res.

[15] Dyson FJ (1982). A model for the origin of life. J. Mol. Evol.

[16] Bollobas B, Rasmussen S (1989). First cycles in random directed graph processes. Discret. Math.

[17] Lifson S (1997). On the crucial stages in the origin of animate matter. J. Mol. Evol.

[18] Szathmary E (2000). The evolution of replicators. Philos. Trans. R. Soc. Lond. B.

[19] Orgel LE (2008). The implausibility of metabolic cycles on the prebiotic earth. PLoS Biol.

[20] Sievers D, von Kiedrowski G (1994). Self-replication of complementary nucleotide-based oligomers. Nature.

[21] Lee DH, Severin K, Ghadiri MR (1997). Autocatalytic networks: The transition from molecular self-replication to molecular ecosystems. Curr. Opin. Chem. Biol.

[22] Ashkenasy G, Jegasia R, Yadav M, Ghadiri MR (2004). Design of a directed molecular network. Proc. Nat. Acad. Sci. USA.

[23] Hayden EJ, von Kieddrowski G, Lehman N (2008). Systems chemistry on ribozyme self-construction: Evidence for anabolic autocatalysis in a recombination network. Angew. Chem. Int. Ed.

[24] Lincoln TA, Joyce GF (2009). Self-Sustained Replication of an RNA Enzyme. Science.

[25] Penny D (2005). An interpretive review of the origin of life research. Biol. Philos.

[26] Hordijk W, Hein J, Steel M (2010). Autocatalytic sets and the origin of life. Entropy.

[27] Dyson FJ (1985). Origins of Life.

[28] Morowitz HJ, Kostelnik JD, Yang J, Cody GD (2000). The origin of intermediary metabolism. Proc. Nat. Acad. Sci. USA.

[29] Smith E, Morowitz HJ (2004). Universality in intermediary metabolism. Proc. Nat. Acad. Sci. USA.

[30] Morowitz HJ, Srinivasan V, Smith E (2010). Ligand field theory and the origin of life as an emergent feature of the periodic table of elements. Biol. Bull.

[31] It was shown in [2] that *P_n_* = 0.50 can be taken as the “transition point” between RAFs not existing at all and RAFs occurring with high probability.

[32] Scott JK, Smith GP (1990). Searching for peptide ligands with an epitope library. Science.

[33] Yamauchi A, Nakashima T, Tokuriki N, Hosokawa M, Nogami H, Arioka S, Urabe I, Yomo T (2002). Evolvability of random polypeptides through functional selection within a small library. Protein Eng.

[34] Bagley RJ (1991). A Model of Functional Self Organization.

[35] Bagley RJ, Farmer JD, Langton CG, Taylor C, Farmer JD, Rasmussen S (1991). Spontaneous Emergence of a Metabolism. Artificial Life II.

[36] Bagley RJ, Farmer JD, Fontana W, Langton CG, Taylor C, Farmer JD, Rasmussen S (1991). Evolution of a metabolism. Artificial Life II.

[37] Andersen IT, Nan L, Kjaersgaard MIS Search for life in catalytic reaction systems. http://www.stats.ox.ac.uk/research/genome/projects/pastprojects.

